# A Case of Dysembryoplastic Neuroepithelial Tumor in an HIV-Positive Adult: Diagnostic Lessons for Clinicians

**DOI:** 10.7759/cureus.101233

**Published:** 2026-01-10

**Authors:** Mariana Lobo, Susana Viana, Andreia Sá Lima, Isabel Monteiro, Carolina M Cerqueira, Frederico Duarte, Luís M Ribeiro, Sara Camões

**Affiliations:** 1 Internal Medicine, Unidade Local de Saúde de Matosinhos - Hospital Pedro Hispano, Matosinhos, PRT; 2 Internal Medicine, Hospital Pedro Hispano, Matosinhos, PRT; 3 Neuroradiology, Hospital Pedro Hispano, Matosinhos, PRT; 4 Infectious Disease, Hospital Pedro Hispano, Matosinhos, PRT; 5 Neurology, Hospital Pedro Hispano, Matosinhos, PRT

**Keywords:** benign pathology, epileptic seizures, hiv aids, neuro radiology, space-occupying brain lesion

## Abstract

Dysembryoplastic neuroepithelial tumor (DNET) is a benign central nervous system neoplasm, usually diagnosed in pediatric patients with epilepsy. We describe a case of a 57-year-old man presenting with a first episode of generalized tonic-clonic seizure and subacute cognitive decline, in whom a multicystic right temporal lobe lesion was detected. Further workup led to a new diagnosis of human immunodeficiency virus (HIV) infection. Comprehensive investigations excluded infectious and malignant etiologies. Review of prior imaging, combined with multidisciplinary input, led to a diagnosis of DNET. Two-year follow-up confirmed radiological stability, and the patient remained seizure-free under antiepileptic treatment. This case highlights the importance of expanding the differential diagnosis of cerebral lesions in HIV-positive adults and utilizing prior imaging to guide clinical decision-making.

## Introduction

Intracranial space-occupying lesions are a frequent and clinically significant finding in patients with HIV infection. The differential diagnosis is broad and commonly includes opportunistic infections, such as cerebral toxoplasmosis and tuberculosis, as well as primary central nervous system lymphoma and other malignancies, particularly in patients with advanced immunosuppression [1,2]. As a result, diagnostic strategies in this population often prioritize infectious and neoplastic etiologies, which may lead to empirical therapy or invasive diagnostic procedures.

Dysembryoplastic neuroepithelial tumor (DNET) is a rare, benign glioneuronal tumor classified as a World Health Organization grade I neoplasm [3]. First described by Daumas-Duport in 1993, DNET is considered a developmental lesion arising from abnormal cortical organization [4]. It typically presents in childhood or early adulthood and is strongly associated with long-standing epilepsy [5]. Adult-onset DNET is uncommon, accounting for a small proportion of reported cases (0.2% of all central nervous system tumors, according to some studies), and may therefore be underrecognized in older patients [6-8].

Neuroimaging plays a central role in the identification of DNET. Characteristic radiological features include a well-circumscribed, cortical-based lesion with a multicystic or "soap-bubble" appearance, hypodensity on computed tomography, hyperintensity on T2-weighted and fluid-attenuated inversion recovery magnetic resonance imaging sequences, and absence of contrast enhancement, significant mass effect, or surrounding edema [5,7-10]. Long-term radiological stability is a key feature that supports a benign diagnosis and may allow conservative management in selected cases [7,8].

The coexistence of DNET and HIV infection has rarely been reported, and no causal association between immunosuppression and DNET pathogenesis has been established. However, the presence of HIV infection may complicate diagnostic reasoning, as benign and non-HIV-related lesions may be overlooked in favor of opportunistic pathology [1,2].

We report a case of DNET in an HIV-positive adult presenting with new-onset seizures, contributing to the limited literature on adult DNET and highlighting the diagnostic complexity of focal brain lesions in patients with HIV infection. This article was previously presented as a meeting abstract at the 16º Encontro do Núcleo de Internos de Medicina Interna on June 30, 2023.

## Case presentation

A 57-year-old man with untreated arterial hypertension and dyslipidemia presented to the emergency department following a first episode of generalized tonic-clonic seizure. He reported four days of progressive memory disturbances, confusion, and disorientation. Physical examination revealed tongue bite marks; neurological examination showed preserved level of consciousness, intact recent and remote memory, normal cranial nerve function, motor strength, sensory examination, coordination, and reflexes, with no focal neurological deficits.

**Figure 1:**
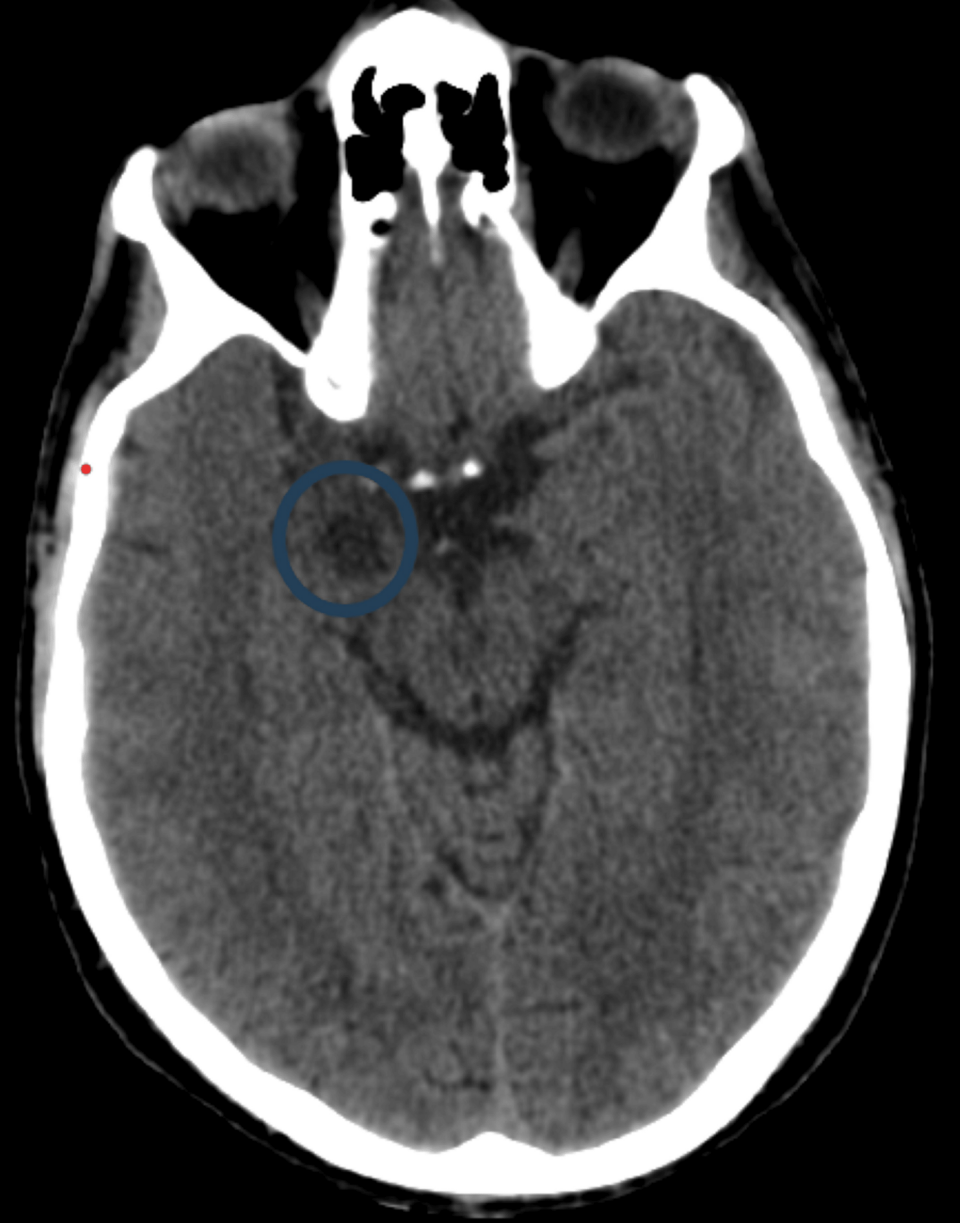
A CT of the brain, taken at the time of admission, revealed a hypodense lesion in the right hippocampus and amygdala (blue circle).

**Figure 2:**
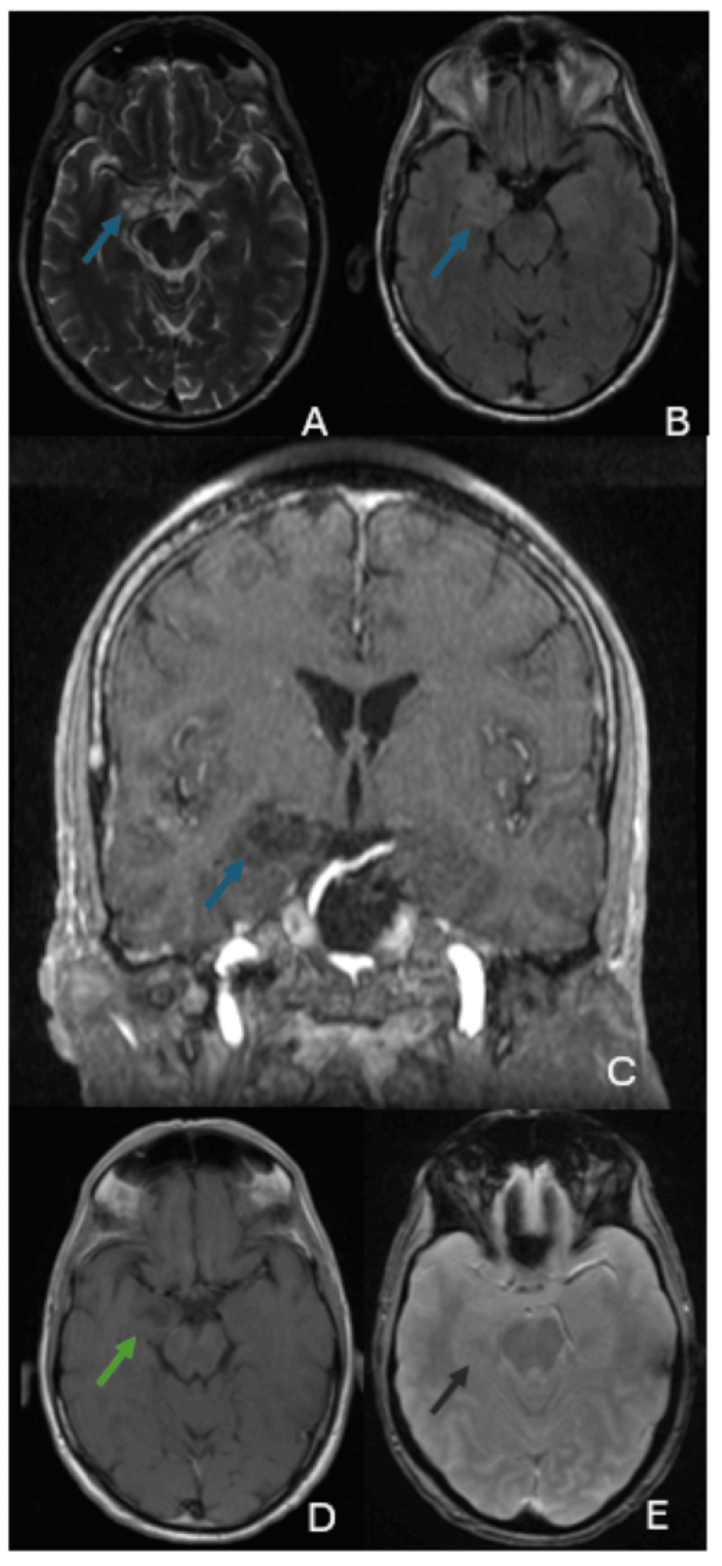
Intra-axial expansile lesion in the right mesial temporal region exhibits hyperintensity on T2-weighted and FLAIR sequences, and hypointensity on T1-weighted images. The images show multiple small cystic components resembling a "soap-bubble" appearance, with no contrast enhancement, vasogenic edema, or signs of hemorrhage or ferromagnetic substance deposition. (A) Axial T2-weighted image; (B) axial FLAIR image; (C) coronal T1-weighted image; (D) axial post-contrast T1-weighted image; (E) axial susceptibility-weighted image.

The initial laboratory evaluation yielded a positive HIV antibody test result and mildly elevated C-reactive protein levels (17 mg/L; normal range: <5 mg/L), but no leukocytosis. Computed tomography (CT) of the brain revealed a hypodense lesion in the right hippocampal-amygdala (Figure 1). Magnetic resonance imaging (MRI) demonstrated a lesion (29x26x22 mm) in the temporal lobe (Figures 2A-2E).

**Figure 3:**
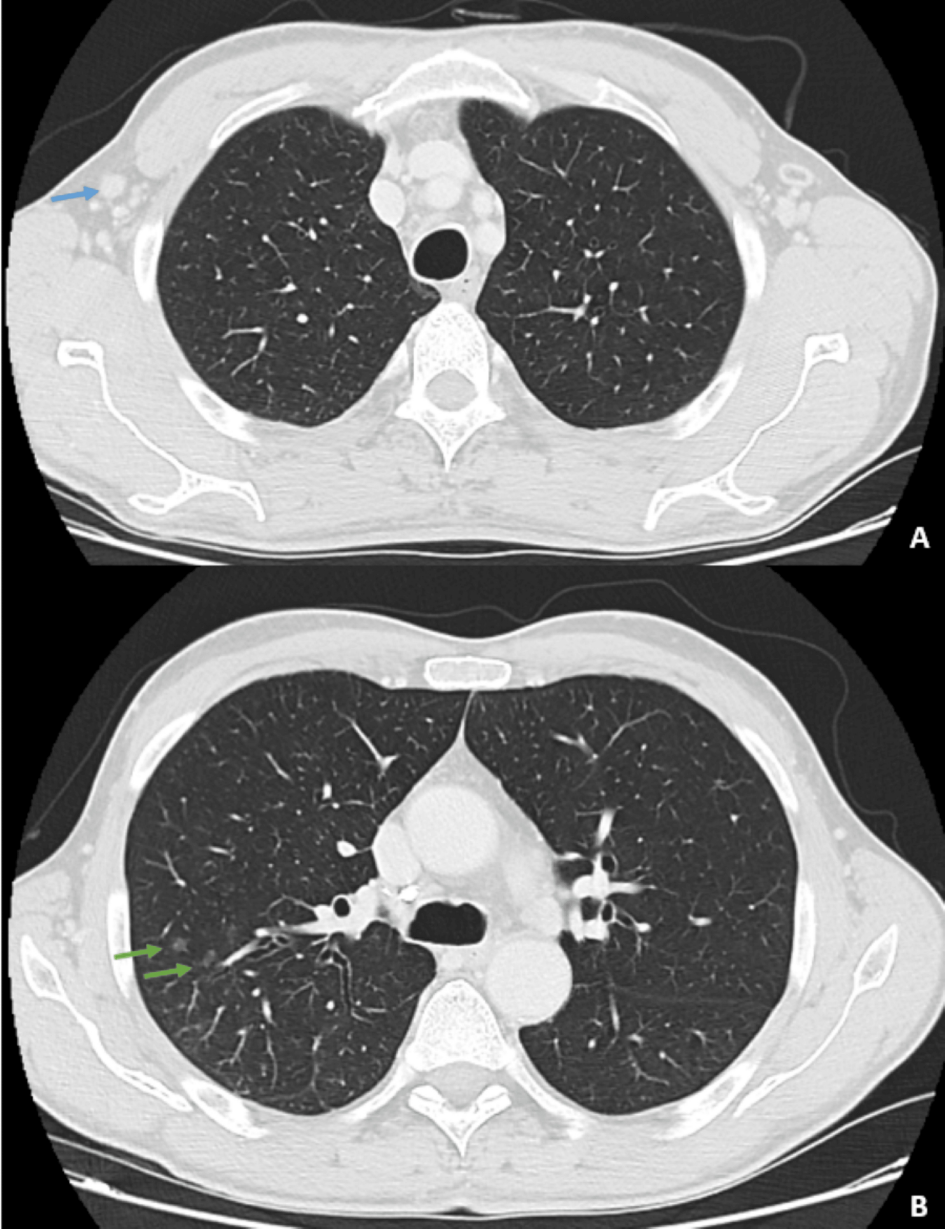
A CT scan of the chest and abdomen showed bilateral axillary lymphadenopathy (indicated by the blue arrows in A) and centrilobular micronodules in the upper lobe of the right lung (indicated by the green arrows in B).

Further workup included chest, abdominal, and pelvis CT scan, which showed bilateral axillary lymphadenopathies and centrilobular micronodules in the right upper pulmonary lobe (Figures 3A, 3B). Lumbar puncture revealed normal opening pressure, 30 cells/µL white blood cells, normal glucose and protein, and negative cryptococcal antigen. Cerebrospinal fluid and serum analyses for *Toxoplasma gondii*, herpesviruses, *Mycobacterium tuberculosis*, and malignant cells were negative. Bronchoscopy with bronchoalveolar lavage was negative for malignant cells and *Pneumocystis jirovecii*.

**Figure 4:**
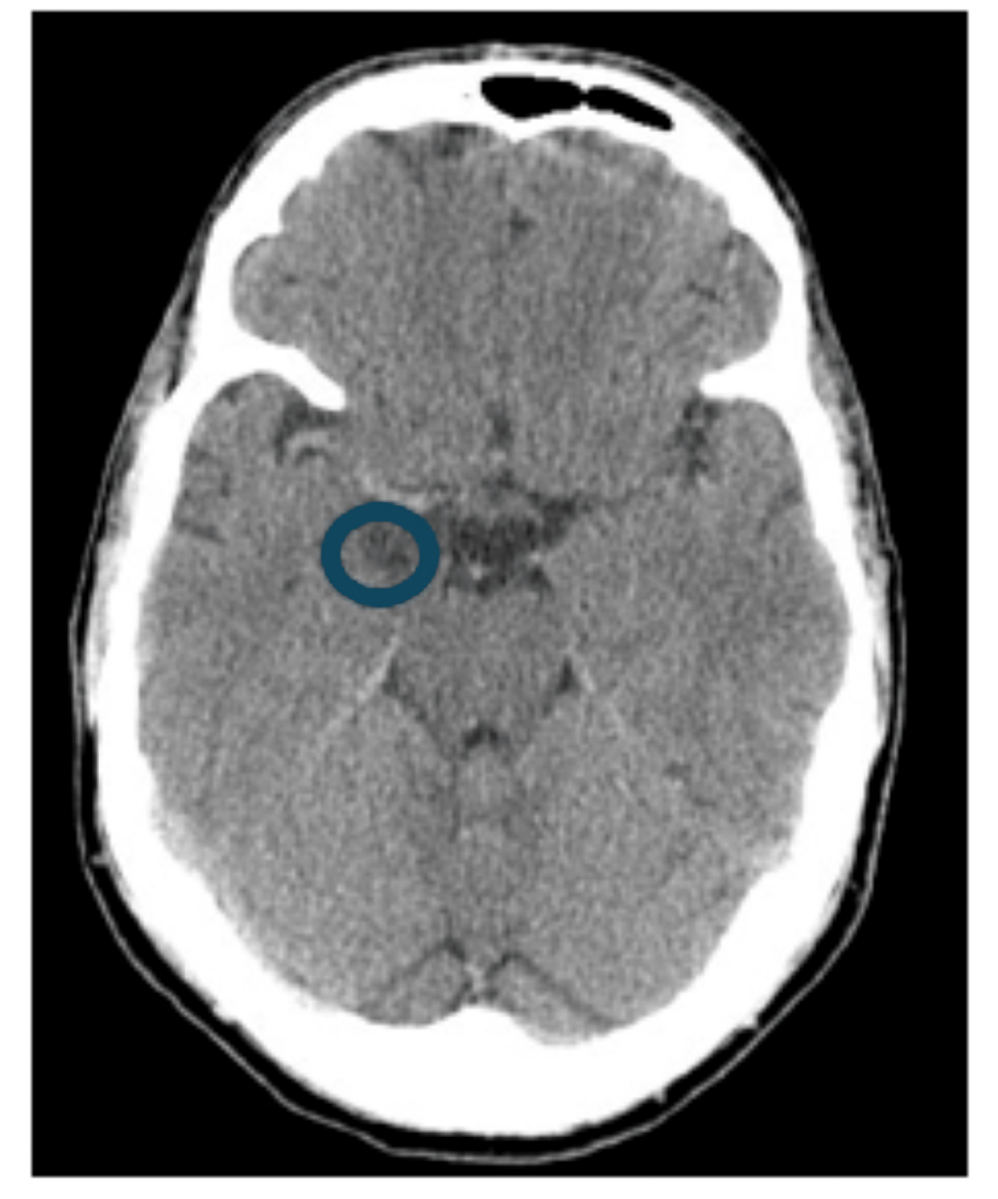
Axial CT image showing a hypodense lesion in the right mesial temporal region (blue circle).

The patient received an 18-day course of empirical antitoxoplasma therapy comprising pyrimethamine, sulfadiazine, and folinic acid. This was discontinued after diagnostic studies consistently failed to detect infection, and imaging remained stable. A retrospective review of a brain CT scan performed six years ago revealed a previously unreported hypodense lesion in the same region (Figure 4). After ruling out an infectious etiology and based on CT and MRI findings indicating a benign etiology (namely DNET), this diagnosis was considered. A multidisciplinary team, including neurosurgeons, considered and supported the provisional diagnosis of DNET, recommending continued radiological surveillance. The patient's seizures were controlled by administering levetiracetam at a dose of 1.5 g every 12 h.

An immunovirological evaluation revealed an HIV-1 infection, with a plasma viral load of 2,493 copies/mL and a CD4+ T-cell count of 369 cells/µL (normal range: 700-1100 cells/µL). The patient was initiated on antiretroviral therapy with dolutegravir and lamivudine, which was well tolerated. Over two years of semestral follow-up, semi-annual MRIs confirmed lesion stability. Attempts to gradually reduce the dose of antiepileptic therapy (to 1 g every 12 h) were unsuccessful due to the recurrence of seizures.

## Discussion

This case highlights the diagnostic challenges posed by focal cerebral lesions in patients with HIV infection, in whom infectious and neoplastic causes are most frequently considered [1,2]. While this approach is appropriate in many clinical settings, it may delay recognition of uncommon but benign entities.

Although DNET is classically diagnosed in children and young adults with epilepsy, cases in adults are becoming more widely recognized and may present with atypical features. Published case studies suggest that seizures are the most common symptom in adults. However, the presentation of cognitive or behavioral changes in our 57-year-old patient is unusual, though consistent with the expanded adult clinical spectrum described in the literature.

Neuroimaging findings were central to the diagnosis in this case. The lesion demonstrated imaging characteristics typical of DNET and remained radiologically stable over time. Longitudinal stability has been repeatedly emphasized as a key feature distinguishing DNET from low-grade gliomas, infectious lesions, and inflammatory processes, which usually demonstrate progression or response to treatment [6-8]. The retrospective identification of a stable lesion on imaging performed several years earlier was particularly valuable and supported a conservative diagnostic strategy without histopathological confirmation.

No association between HIV infection and DNET has been established, and their coexistence in this patient was most likely incidental. DNET is considered a developmental tumor rather than an acquired neoplasm related to immunosuppression [10]. Nevertheless, HIV infection significantly influenced the diagnostic approach, initially favoring an infectious etiology, as commonly reported in patients with intracranial lesions and HIV [1,2]. This case illustrates the risk of diagnostic anchoring in this population and underscores the importance of integrating imaging characteristics and clinical evolution into decision-making.

Surgical resection is recommended for patients with refractory epilepsy and is associated with favorable seizure outcomes [6,8,10]. However, conservative management with antiepileptic therapy and radiological surveillance is an accepted approach in selected patients with stable lesions and adequate seizure control [7,8]. The favorable clinical and radiological course observed in our patient supports this strategy.

In comparison with previously published reports, this case is notable for the advanced age at presentation, the diagnostic context of newly diagnosed HIV infection, and the decisive role of historical imaging. It adds to the limited literature on adult-onset DNET and emphasizes the need for individualized, multidisciplinary assessment of focal brain lesions in HIV-positive patients.

## Conclusions

This case demonstrates that HIV-positive patients presenting with new-onset seizures and space-occupying lesions in the brain may have benign causes, such as DNET. In this case, the focus is on the diagnostic difficulties in evaluating a brain space-occupying lesion in an HIV-positive patient due to various potential causes. HIV was primarily a complicating factor, and a detailed review of prior test results played a crucial role in achieving the diagnosis, helping to prevent unnecessary invasive procedures and to ensure suitable management.
